# Analysis of genes characterizing chronic thrombosis and associated pathways in chronic thromboembolic pulmonary hypertension

**DOI:** 10.1371/journal.pone.0299912

**Published:** 2024-03-07

**Authors:** Shizhang Zhan, Liu Wang, Wenping Wang, Ruoran Li

**Affiliations:** 1 Bengbu Medical College, Bengbu, China; 2 Xuzhou Central Hospital, Xuzhou, China; BSMMU: Bangabandhu Sheikh Mujib Medical University, BANGLADESH

## Abstract

**Purpose:**

In chronic thromboembolic pulmonary hypertension (CTEPH), fibrosis of thrombi in the lumen of blood vessels and obstruction of blood vessels are important factors in the progression of the disease. Therefore, it is important to explore the key genes that lead to chronic thrombosis in order to understand the development of CTEPH, and at the same time, it is beneficial to provide new directions for early identification, disease prevention, clinical diagnosis and treatment, and development of novel therapeutic agents.

**Methods:**

The GSE130391 dataset was downloaded from the Gene Expression Omnibus (GEO) public database, which includes the full gene expression profiles of patients with CTEPH and Idiopathic Pulmonary Arterial Hypertension (IPAH). Differentially Expressed Genes (DEGs) of CTEPH and IPAH were screened, and then Kyoto Encyclopedia of Genes and Genomes (KEGG) and Gene Ontology (GO) functional enrichment analyses were performed on the DEGs; Weighted Gene Co-Expression Network Analysis (WGCNA) to screen the key gene modules and take the intersection genes of DEGs and the key module genes in WGCNA; STRING database was used to construct the protein-protein interaction (PPI) network; and cytoHubba analysis was performed to identify the hub genes.

**Results:**

A total of 924 DEGs were screened, and the MEturquoise module with the strongest correlation was selected to take the intersection with DEGs A total of 757 intersecting genes were screened. The top ten hub genes were analyzed by cytoHubba: IL-1B, CXCL8, CCL22, CCL5, CCL20, TNF, IL-12B, JUN, EP300, and CCL4.

**Conclusion:**

IL-1B, CXCL8, CCL22, CCL5, CCL20, TNF, IL-12B, JUN, EP300, and CCL4 have diagnostic and therapeutic value in CTEPH disease, especially playing a role in chronic thrombosis. The discovery of NF-κB, AP-1 transcription factors, and TNF signaling pathway through pivotal genes may be involved in the disease progression process.

## 1. Introduction

CTEPH remains an unresolved health problem worldwide due to its high rates of disability and mortality [1]. It is currently believed that the main pathogenesis of CTEPH is divided into two parts, on the one hand, CTEPH is a distant complication of acute pulmonary thromboembolism (APTE), in which the formation of chronic thrombus in the lumen of the blood vessel leads to vascular obstruction, which in turn leads to pulmonary hypertension [2]. It is also believed that chronic thrombus is closely related to factors such as the blood system or immune system and fibrinolytic system that cause the thrombus to be difficult to regress [3–5]. On the other hand, vascular injury and progressive pulmonary vascular remodeling are the pathological basis and important factors in the disease progression of CTEPH [6–8]. Therefore, it is believed that CTEPH is a class of pulmonary hypertension caused by the combined effect of dual pulmonary vascular diseases [3]. A new approach has emerged in group 4 of pulmonary hypertension, where the concept of chronic thromboembolic disease without PH (CTED) has gained popularity [9]. Due to the high degree of similarity between CTEPH and IPAH in terms of histologic changes such as vascular injury, pulmonary artery remodeling, and right ventricular remodeling, and IPAH has not been reported to have thrombus formation [6, 10, 11]. Therefore, in order to find the characteristic genes that cause chronic thrombosis in CTEPH disease more precisely, we mainly compared the samples of CTEPH and IPAH, aiming to provide theoretical references for the search of CTEPH biomarkers and the development of new target diagnostic and therapeutic directions.

Gene expression microarrays are now commonly used in gene expression profiling studies, providing new ways to explore key genes in diseases, as well as providing potential value for developing new directions in clinical diagnosis and treatment. Based on this, this study proposed to download the original microarray dataset GSE130391 (14 CTEPH patient samples and 4 IPAH samples) through the GEO database. The DEGs of CTEPH and IPAH were screened using the package in R software (version 4.2.3), and the DEGs were analyzed for GO and KEGG pathway enrichment. The WGCNA algorithm was then used to filter the high correlation modules to take intersections with DEGs. Finally, protein-protein interaction (PPI) network was constructed using STRING online database to analyze the proteomic associations, and using cytoHubba analysis, the top 10 hub genes with the highest degree were found. The molecular mechanisms of the hub genes and the associated pathways were explored for their impact on disease.

## 2. Information and methods

### 2.1. Information

Genetic data were downloaded from the GEO database (GSE130391), which included 14 samples of CTEPH, 4 samples of IPAH, and 4 normal samples. The full gene expression profiles of the 14 CTEPH samples and 4 IPAH samples were subjected to data analysis.

### 2.2. Methods

#### 2.2.1. Screening of DEGs

The ggplot2, limma, and pheatmap packages were used to identify DEGs between CTEPH samples as well as to plot differential gene volcano maps and heat maps. t-tests were utilized to calculate p-values for DEGs, with adjusted p-values of 0.05 and |logFC|>1.0 serving as screening criteria. The limma package is a linear model-based tool for differential gene expression analysis, which exhibits good noise resistance and a low false positive rate. It demonstrates strong resistance to noise by utilizing a Bayesian approach to model noise, making it particularly effective when dealing with high-dimensional data, small sample sizes, and increased levels of noise. Additionally, the limma package employs adjusted P-values to control the error rate of multiple comparisons, thereby effectively reducing the occurrence of false positives. For creating heatmaps, the pheatmap package is commonly employed. Heatmaps are frequently used to visually represent matrix-type data, where different colors indicate varying magnitudes of values. ggplot2, on the other hand, is the most widely used package for data visualization. It offers the advantage of a clear syntax and a high degree of customizability, enabling the creation of professional and informative graphs.

#### 2.2.2. KEGG, GO enrichment analysis of DEGs

KEGG and GO enrichment analysis of DEGs was performed using clusterProfiler, org.Hs.eg.db, enrichplot, and ggplot2 packages. The pvalueFilter was set to 0.05 and qvalueFilter to 1. The clusterProfiler package is utilized for conducting biological function enrichment analyses to aid in the identification of enriched functions in a gene set. It also offers comprehensive graphical representation and interpretation of the results. The org.Hs.eg.db package, based on Homo sapiens (human), serves as a gene annotation database to facilitate the interpretation and annotation of biological significance within a gene set. Meanwhile, the enrichplot package provides a variety of graphs and charts for visualizing functional enrichment analysis results, effectively presenting the significance and relevance of these findings.

#### 2.2.3 Application of WGCNA to screen key gene modules

Co-expression networks were constructed in the GSE130391 cohort using the weighted gene co-expression network analysis (WGCNA) method. The advantage of WGCNA lies in its ability to construct co-expression networks and identify gene modules with similar expression patterns from gene expression data, thereby revealing gene interactions and biological relevance. The WGCNA package calculates the soft threshold power as well as the neighboring relationships, converts the neighboring matrix to topological overlap matrix (TOM), and calculates the corresponding variability for hierarchical clustering analysis. To find co-expressed genes, a dynamic tree-cutting approach with a module size of 50 was used. Key modules were identified by evaluating the relationship between gene modules and CTEPH using the gene significance (GS) value and the module membership (MM) value. The next step involved screening highly correlated modules and intersecting them with differentially expressed genes (DEGs).

#### 2.2.4 Construction of PPI network and identification of hub genes

The PPI network of DEGs was predicted using the Search Tool for Online Interacting Genes (STRING) database with the minimum required interaction score set: highest confidence (0.900) and hide disconnected nodes in the network. STRING not only provides comprehensive data on gene and protein interactions, but also offers a wide range of analysis and visualization tools. Finally, cytoHubba analysis revealed the top 10 hub genes with the highest degree. CytoHubba, a plug-in for Cytoscape, provides multiple node importance algorithms, flexible customizability, results visualization, and integration with Cytoscape, enabling comprehensive gene/protein network analysis and identification of key nodes in the network.

### 2.3 Observation indicators

(i) analysis of DEGs between normal and disease groups; (ii) application of GO and KEGG to analyze DEGs; (iii) application of WGCNA to screen key gene modules; (iv) scoring of PPI networks and pivotal genes; (v) molecular mechanisms and major enrichment pathways of pivotal genes.

### 2.4 Statistical methods

All data in this study were analyzed using R software (version 4.2.3), Cytoscape software (version 3.9.1), and the STRING database (https://cn.string-db.org). p < 0.05 was considered statistically significant.

## 3 Results

### 3.1 Analyzing DEGs between CTEPH group and IPAH group

**Fig 1:**
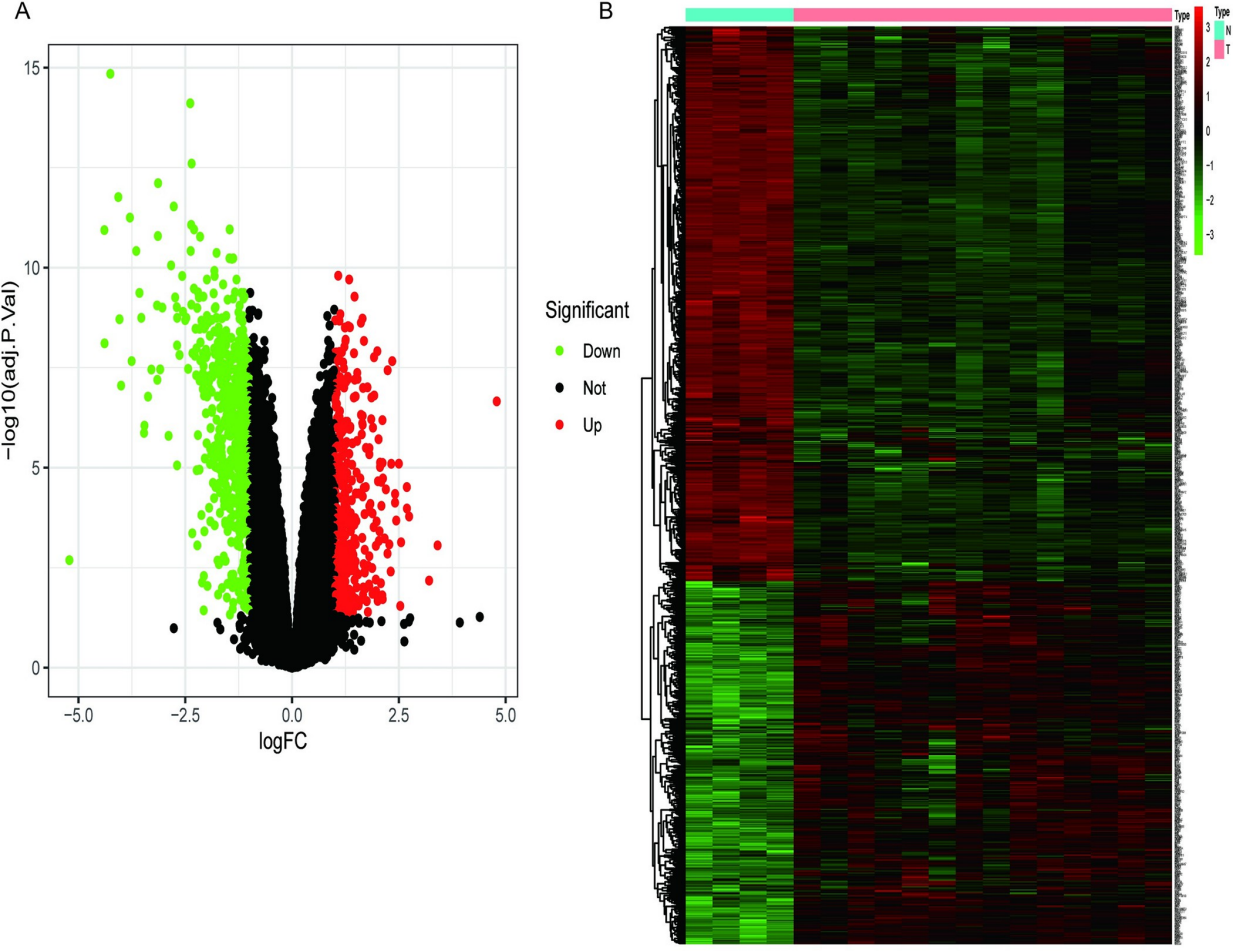
DEGs between CTEPH group and IPAH group. (A) Volcano plot illustrating the differential expression of genes between the CTEPH and IPAH groups. Red dots represent up-regulated genes, while green dots represent down-regulated genes. (B) Heat map of differential gene expression between CTEPH and IPAH groups.

The DEGs of CTEPH patients in the observation group and IPAH patients in the control group were analyzed by the R program Limma package, and a total of 924 DEGs were screened to generate volcano plots (Fig 1A) and heat maps (Fig 1B).

### 3.2 KEGG and GO enrichment analysis of DEGs

**Fig 2:**
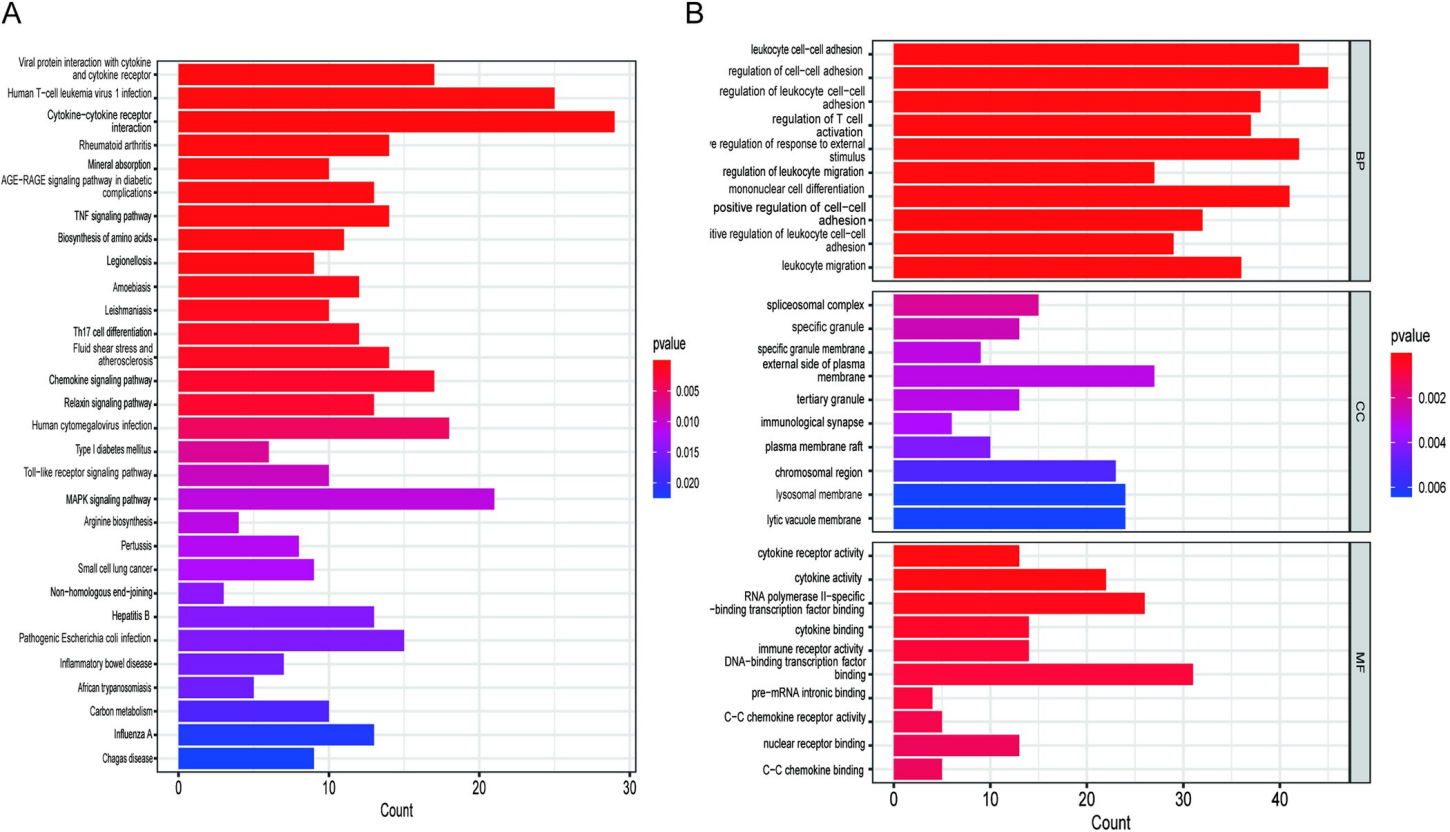
Functional enrichment analysis of DEGs. (A) KEGG enrichment analysis of DEGs; (B) GO enrichment analysis of DEGs.

KEGG results showed that the first five major pathways of enrichment were Cytokine−cytokine receptor interaction、Human T−cell leukemia virus 1 infection、TNF signaling pathway、Rheumatoid arthritis、Viral protein interaction with cytokine and cytokine receptor (Fig 2A). Biological Process (BP) results showed that the top three were eukocyte cell−cell adhesion、regulation of cell−cell adhesion and positive regulation of response to external stimulus(Fig 2B). Cellular Component (CC) results showed that the top three positions were external side of plasma membrane、spliceosomal complex and lysosomal membrane(Fig 2B). Molecular Function (MF) results showed that DNA−binding transcription factor binding、RNA polymerase II−specific DNA−binding transcription factor binding、cytokine activity (Fig 2B).

### 3.3 Application of WGCNA to screen key gene modules

**Fig 3:**
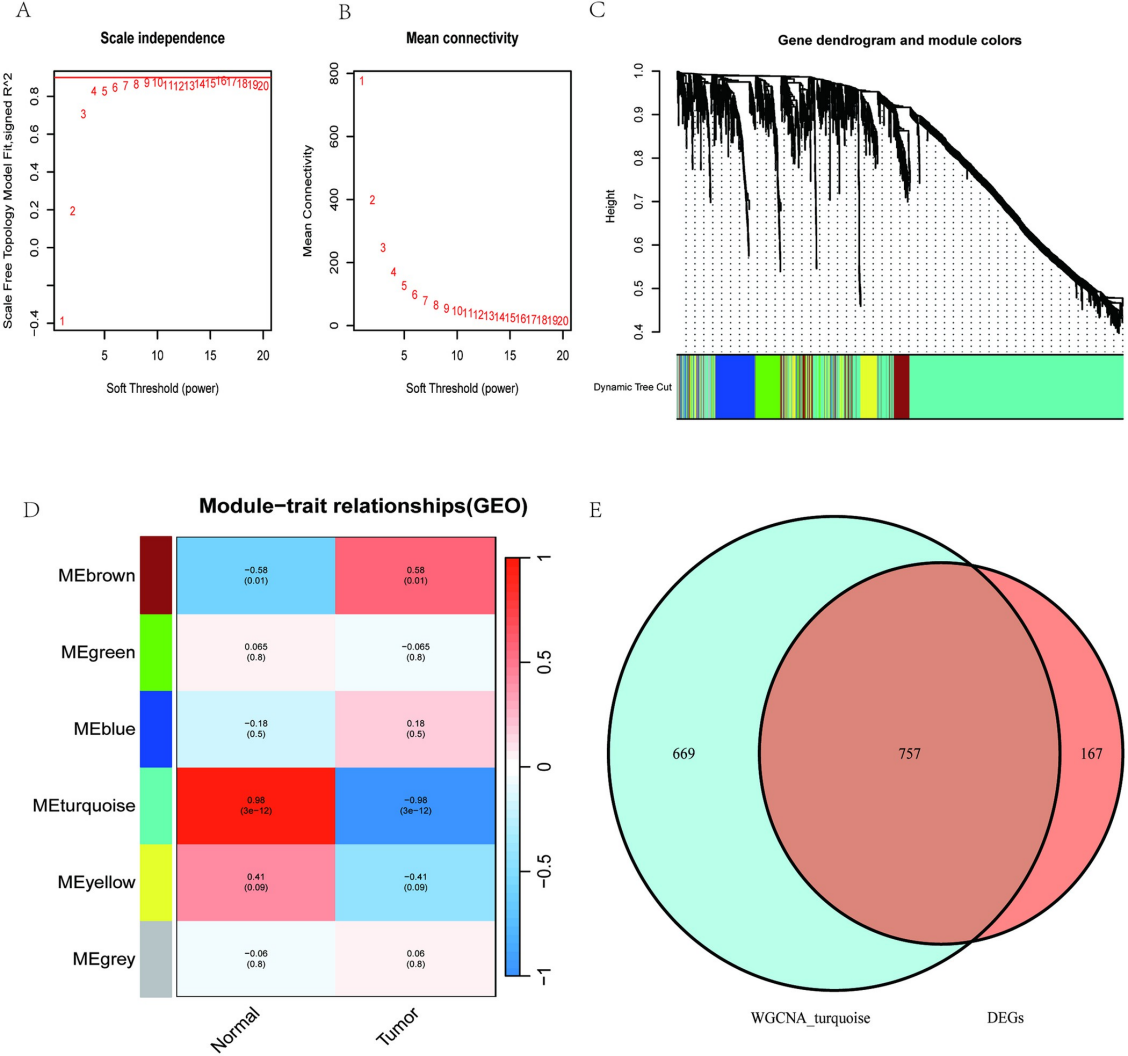
WGCNA identification of key gene modules. (A) soft threshold of WGCNA; (B) average connectivity of WGCNA; (C) cluster dendrogram of WGCNA; (D) key gene module map of WGCNA; (E) MEturquoise module genes intersected with DEGs taken.

The R software WGCNA package was used to analyze the CTEPH group and IPAH group, and the scale-free co-expression network was established, and the soft threshold power was determined to be 9, and the scale-free index was 0.85, and a total of 6 key gene modules were screened, and the correlation between each module and CTEPH was calculated, and The results indicated a significant correlation between the MEturquoise module and CTEPH (cor = -0.98, P < 0.05). In this study, the MEturquoise module contained 1426 genes, and 757 intersecting genes were obtained by taking the intersection of MEturquoise module genes and DEGs, as detailed in Fig 3.

### 3.4. set up PPI network as well as cytoHubba to analyze the intersection genes of MEturquoise module genes with DEGs

**Fig 4:**
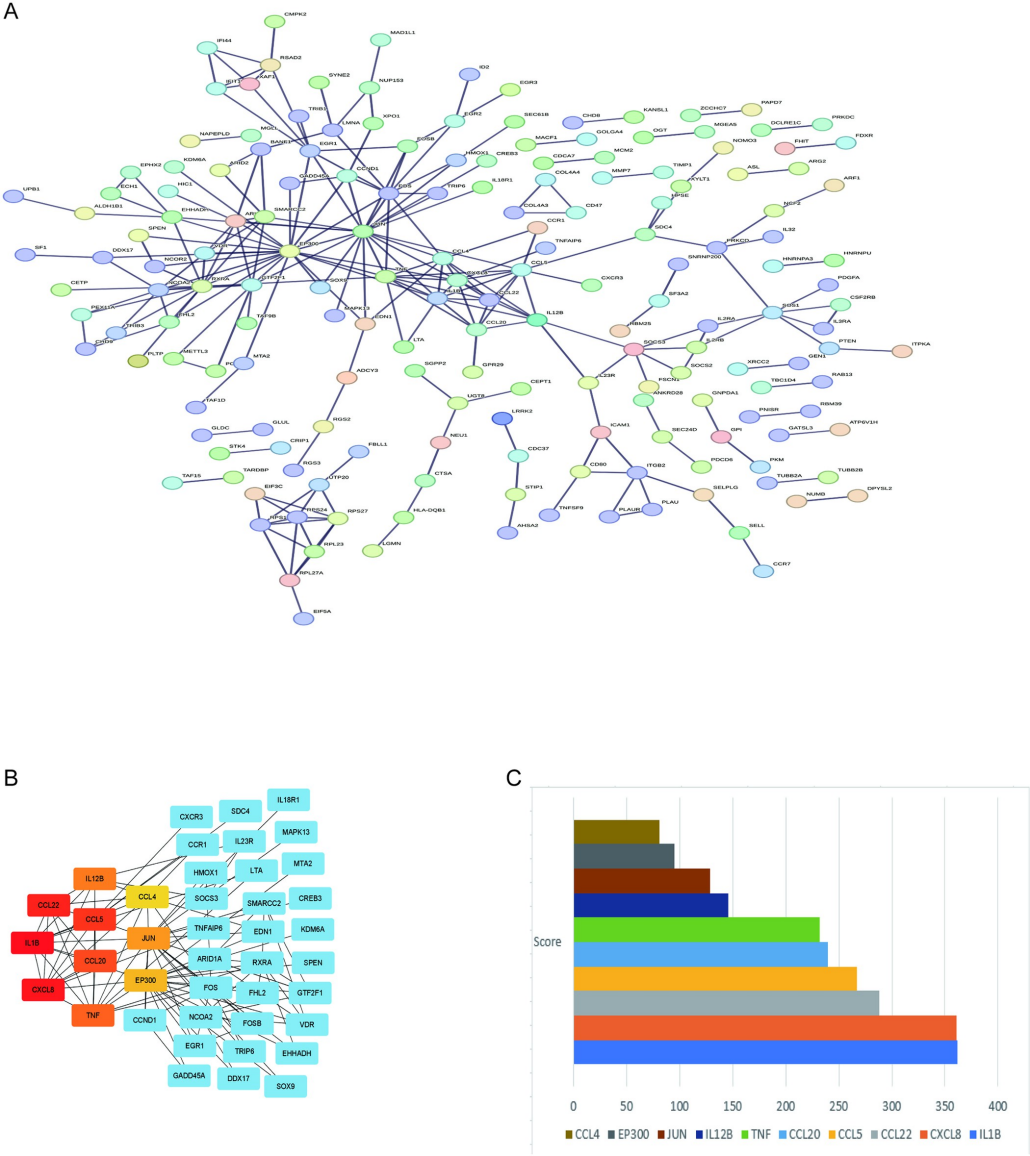
PPI network and hub genes. (A) PPI network constructed by STRING database; (B) top 10 Hub genes screened between CTEPH and control IPAH; (C) histogram of the top 10 Hub gene scores screened between CTEPH and control IPAH.

The PPI network between intersecting genes was constructed by STRING database. Set the minimum required interaction score:0.900; remove all individual or isolated nodes to construct an integrated network as in Fig 4A. The top 10 Hub genes were identified by cytoHubba analysis, with the shade of red representing the degree of importance as in Fig 4B. Therefore, according to the order of the hierarchical order, they are IL-1B, CXCL8, CCL22, CCL5, CCL20, TNF, IL-12B, JUN, EP300, CCL4 (Fig 4C).

### 3.5 Hub gene expression and pathway relationships

**Fig 5:**
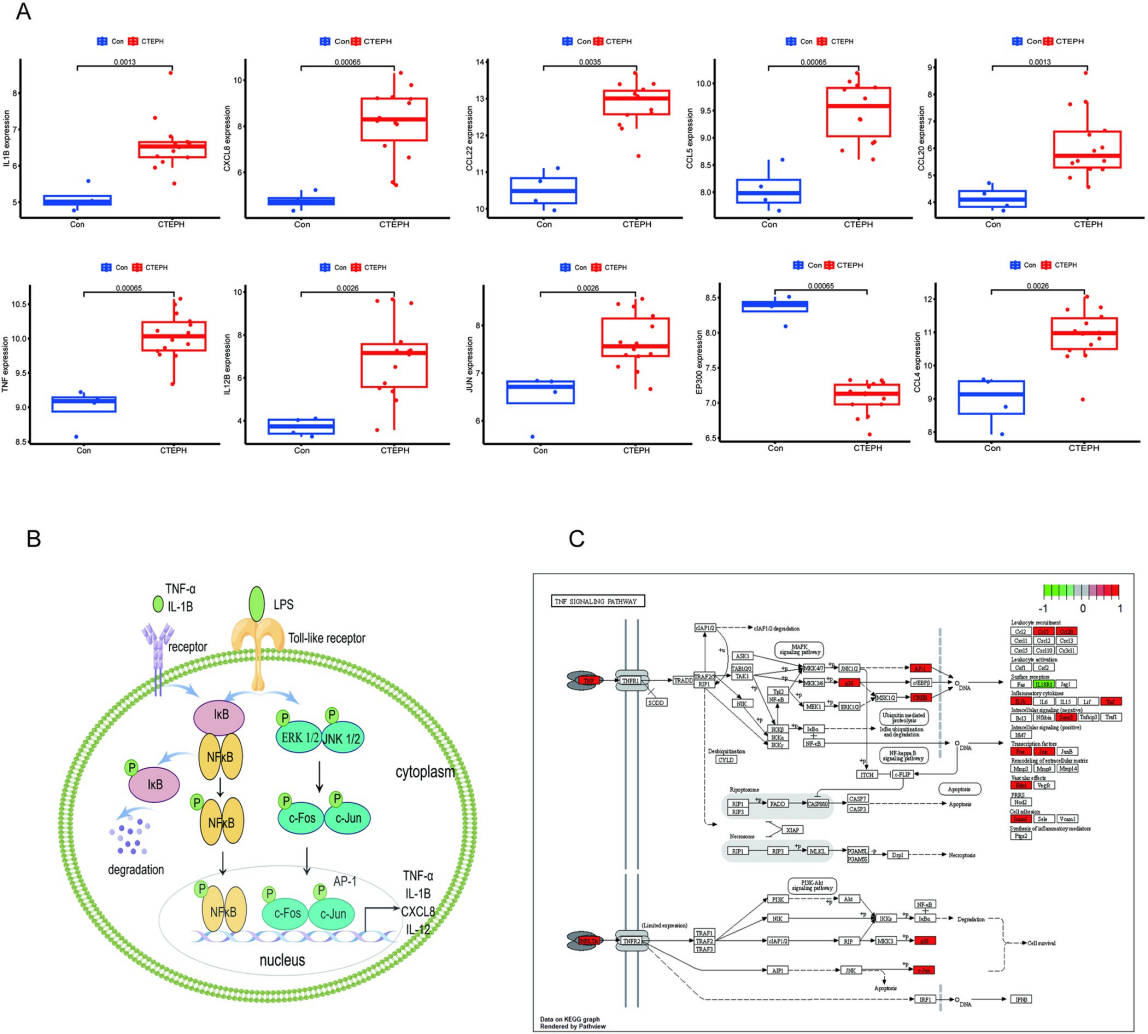
Differential expression of characterized genes and analysis of related pathways. (A) Gene expression of IL-1B, CXCL8, CCL22, CCL5, CCL20, TNF, IL-12B, JUN, and CCL4 in the disease group CTEPH and the control group IPAH; (B) Transcriptional schematic diagrams of NF-Κb, AP-1, and others. (C) Diagram of TNF signaling pathway.

Compared with the IPAH population, IL-1B, CXCL8, CCL22, CCL5, CCL20, TNF, IL-12B, JUN, and CCL4 were highly expressed in CTEPH patients, suggesting that the above nine characterized genes were expressed higher in CTEPH disease than in IPAH patients; the opposite was true for EP300 (Fig 5A). In addition, the expression relationship of the characterized genes in cells was mapped according to existing studies [12, 13] (Fig 5B). Finally, based on the expression and pathway enrichment of the characterized genes, it was found that the characterized genes were mostly expressed in the TNF signaling pathway (Fig 5C).

## 4. Discussion

The pathological features of CTEPH are mainly the presence of obstructive fibrotic thromboembolic material in the great vessels and progressive pulmonary vascular remodeling [14, 15]. In recent years, research on CTEPH has largely centered on these two items. The risk factors that have been demonstrated include inflammation and infection, abnormalities of the coagulation system, abnormalities of fibrinolysis, abnormalities of platelet function, cancer, blood group, myofibroblasts, impaired angiogenesis, and impaired small vessels [16]. Therefore, the therapeutic regimens developed around the pathological features and related risk factors are constantly being updated, and their treatments mainly include basic therapy (long-term anticoagulation and home oxygen therapy, etc.), surgery (pulmonary endarterectomy, balloon pulmonary arterioplasty, etc.), and pharmacological treatments (soluble guanylate cyclase-stimulating agent leucovorin, etc.) [16–19]. However, there are still cases such as not meeting the indications for surgery or ineffective drug therapy, and the average 5-year survival rate of CTEPH after treatment is about 53–69% [20–22]. Therefore, the search for CTEPH biomarkers and the development of new targets for diagnosis and treatment are urgent. In this study, we determined whether the pivotal genes are associated with chronic thrombosis by comparing the differentially expressed genes in the pulmonary vasculature in CTEPH and IPAH samples. In this study, the results showed that 924 significant DEGs were derived by screening, including 364 up-regulated and 560 down-regulated genes. The results of enrichment analysis showed that most of the differential genes were associated with cytokines, suggesting that cytokines are crucial in the disease progression of CTEPH. Moreover, GO enrichment analysis suggested that the major roles of the differential genes were transcription factor binding, cytokine activity and cell adhesion, and it is now well established that disease progression is associated with these molecular functions or biological processes. It further validates that our screened differential genes and the disease are correlated.

In this study, we analyzed the GEO dataset by WGCNA to get the high correlation module genes and DEGs to take the intersection, and identified 10 pivotal genes including IL-1B, CXCL8, CCL22, CCL5, CCL20, TNF, IL-12B, JUN, EP300, and CCL4 by STRING online network and cytoHubba analysis. Studies have shown that pulmonary thromboembolism is closely related to inflammatory factors and chemokines, etc., and inflammatory factors such as IL-1B, CXCL8, and TNF-α are expressed at high levels in patients with pulmonary thromboembolism, and individuals with high-risk pulmonary embolism have higher levels of inflammatory factors than individuals with intermediate- and low-risk pulmonary embolism [23]. Adding IL-1B to whole blood can cause hypercoagulation of blood and also fibrin aggregation, which reduces the rate of clot dissolution [24]. Inflammatory factors such as IL-1B may be important in causing pulmonary vasoconstriction and reduced pulmonary blood flow in a rabbit model of acute pulmonary thromboembolism [25]. In a mouse tumor model, IL-1B may promote thrombosis through the regulation of neutrophil colony-stimulating factor [26].NOD-like Receptor Thermal Protein domain Associated Protein 3 (NLRP3) activated under hypoxia NLRP3 is activated under hypoxia, which promotes IL-1B expression, contributes to venous thrombosis, and thus induces platelet activation [27]. Moreover, it has been pointed out [28] that IL-1B-binding antibodies help to reduce the inflammatory response and risk of deep vein thrombosis.NicolaPotere et al. [29] found that patients with novel coronavirus pneumonia generally had significant inflammatory response and coagulation dysfunction, and concluded that the activation of NLRP3 and the release of IL-1β after novel coronavirus pneumonia infection may exacerbate the lung injury and induce a hypercoagulable state and suggested that the IL-1 family is a driver of excessive inflammation and injury in this disease.CXCL8 can be produced and released by a variety of cells in response to endogenous or exogenous pro-inflammatory stimuli [30]. Moreover, the production of CXCL8 is directly related to the thrombin-antithrombin (TAT) complex [31]. It was noted [32] that the addition of CXCL8 to the blood of normal subjects resulted in a significant increase in clot formation and an increase in cross-linked fibrin. It is also believed that elevated levels of CXCL8 may lead to the emergence of small thrombi, thereby increasing the risk of small vessel occlusion.Emily Bontekoe et al. concluded that high expression of CXCL8 leads to thrombosis and may be an important indicator of the severity of embolism [33].TNF is a major regulator of inflammation and a key player in the cytokine network, and it has been demonstrated that TNF plays an important role in the formation of thrombus [34]. The role of TNF in the formation of thrombus is not only a major regulator of inflammation but also a key participant in the cytokine network. TNF alters coagulation factor VIII and fibrinogen, activates platelets, inhibits protein C activation and tissue fibrinolytic enzyme secretion, and secretes inhibitors of fibrinogen activation, which hinders fibrinolysis and promotes blood coagulation in patients [32, 35]. There is no evidence for the association of CCL22, CCL5, CCL20, and CCL4 chemokines with events such as thromboembolism and thrombosis [32, 35].CCL22, CCL5, CCL20, and CCL4 chemokines have no significant role in thrombosis and thrombophilia. significant correlation with events such as thromboembolism, but they are involved in migration, adhesion, degranulation and apoptosis of inflammatory cells, which in turn cause tissue inflammatory responses [36, 37]. In contrast, FOS/JUN dimer is the predominant form of activator protein-1 (AP-1) in most cells and is involved in inducing thrombus formation and expression of pro-inflammatory factors [38].

Studies have pointed out [39, 40] that chronic thrombosis, chronic inflammatory stimuli and high expression of inflammatory cytokines may be important factors contributing to endothelial cell dysfunction in pulmonary arteries in CTEPH. Endothelial cell dysfunction can cause anticoagulant dysfunction and may lead to additional in situ thrombosis. Endothelial cells in patients with CTEPH are functionally impaired due to high production of inflammatory cytokines and chemokines, which are involved in thrombosis [41, 42]. However, some scholars argue that the pulmonary vascular endothelium plays a crucial role in connecting the blood circulation and the sub-tissues of the vascular endothelium. With the endothelial dysfunction of the pulmonary artery, inflammatory factors, chemokines, and adhesion molecules in the blood can directly enter the vascular subendothelial tissues, which leads to the value-added, differentiation, and migration of the vascular smooth muscle cells, thus causing vascular remodeling [43–46]. This shows that cytokines such as IL-1B, CXCL8, IL-12, TNF, CCL22, CCL20, CCL5, CCL4, etc. are involved in the disease development of CTEPH.

By combining analysis of pivotal genes and related pathways, we observed a significant overlap between the pivotal genes and the downstream cytokines of NF-κB (nuclear factor kappa-B) and AP-1 transcription factors. This suggests a close association between transcription factors such as NF-κB, AP-1, and other transcription factors with the progression of the disease. This finding is consistent with the study conducted by Valérie F.E.D. Smolders et al, which also highlighted the involvement of IL-1B, CXCL8, and other factors in the progression of CTEPH [47]. Minxia Yang et al. also concluded through clinical studies that inflammatory response and inflammatory factors play an important role in the pathogenesis of CTEPH through the inflammation-coagulation-thrombosis cycle [48]. Meanwhile, enrichment analysis yielded that most of the hub genes were expressed in the TNF signaling pathway. The TNF signaling pathway transmits signals through two receptors, TNFR1 and TNFR2, which are pro-inflammatory, while TNFR2 is associated with tissue repair, growth regulation, and differentiation. The TNF signaling pathway exerts pro-inflammatory, pro-apoptotic, and pro-value-added roles in tissues, which may be involved in the progression of disease. Frank Langer et al. highlighted the crucial role of the inflammatory response and inflammatory factors in the progression of chronic thromboembolic pulmonary hypertension (CTEPH). They compared the changes in the levels of inflammatory factors in the blood before and after pulmonary thromboendarterectomy (PTE) in CTEPH patients. They observed that inflammatory factors such as TNF-α, which were elevated before the surgery, returned to normal rapidly after the surgical intervention [49]. In the future, it is essential to perform functional validation and mechanistic studies, investigate regulatory and interaction networks associated with identified key genes and related pathways to provide novel directions for disease research, drug development, and therapeutic strategies. Theoretical validation work can be accomplished by utilizing cellular models, animal models, or in vitro experiments to uncover the basis for comprehensive clinical and translational research. Additionally, it is crucial to explore the potential value of identified key genes and pathways as biomarkers to facilitate the development of personalized medicine and precision medicine.

This study also has several limitations. Firstly, the data for the study were obtained from public databases, and no cell models, animal models, or in vitro experiments were conducted to measure the expression of relevant genes and pathways. Secondly, the sample size was relatively small, which may introduce selective bias and should be expanded to validate the current findings. Finally, the screened pivotal genes indicate an inflammatory response. While inflammatory response and inflammatory factors are recognized as important influences on CTEPH, it is necessary to exclude interfering factors and confirm them through more clinical and in vitro studies.

## 5 Conclusion

In conclusion, bioinformatics analysis showed that the disease progression of CTEPH is closely related to hub genes (IL-1B, CXCL8, CCL22, CCL5, CCL20, TNF, IL-12B, JUN, EP300, CCL4). Meanwhile, the above hub genes can cause thrombus formation, mechanization, and endothelial cell dysfunction, which are involved in chronic thrombus formation and arterial vascular remodeling. In addition, NF-κB, AP-1 transcription factor and TNF signaling pathway, which are highly overlapping with the hub genes, may be involved in the disease progression.

## Supporting information

S1 File (TXT)

S2 File (TXT)

S3 File (TXT)

S4 File (TXT)

S5 File (TSV)

S6 File (CSV)
